# Variable detection of Omicron-BA.1 and -BA.2 by SARS-CoV-2 rapid antigen tests

**DOI:** 10.1007/s00430-022-00752-7

**Published:** 2022-11-12

**Authors:** Andreas Osterman, Irina Badell, Christopher Dächert, Nikolas Schneider, Anna-Yasemin Kaufmann, Gamze Naz Öztan, Melanie Huber, Patricia M. Späth, Marcel Stern, Hanna Autenrieth, Maximilian Muenchhoff, Alexander Graf, Stefan Krebs, Helmut Blum, Ludwig Czibere, Jürgen Durner, Lars Kaderali, Hanna‑Mari Baldauf, Oliver T. Keppler

**Affiliations:** 1 Max Von Pettenkofer Institute and Gene Center, Virology, National Reference Center for Retroviruses, LMU München, Munich, Germany; 2 German Center for Infection Research (DZIF), Partner Site Munich, Munich, Germany; 3 COVID-19 Registry of the LMU Munich (CORKUM), University Hospital, LMU München, Munich, Germany; 4 Laboratory for Functional Genome Analysis, Gene Center, LMU München, Munich, Germany; 5 Labor Becker MVZ GbR, Munich, Germany; 6 Department of Conservative Dentistry and Periodontology, University Hospital, LMU München, Munich, Germany; 7 Institute of Bioinformatics, University Medicine Greifswald, Greifswald, Germany

**Keywords:** SARS-CoV-2, VOC, Omicron, BA.1, BA.2, RAT, Nucleocapsid protein, Sensitivity, Specificity

## Abstract

During 2022, the COVID-19 pandemic has been dominated by the variant of concern (VoC) Omicron (B.1.1.529) and its rapidly emerging subvariants, including Omicron-BA.1 and -BA.2. Rapid antigen tests (RATs) are part of national testing strategies to identify SARS-CoV-2 infections on site in a community setting or to support layman’s diagnostics at home. We and others have recently demonstrated an impaired RAT detection of infections caused by Omicron-BA.1 compared to Delta. Here, we evaluated the performance of five SARS-CoV-2 RATs in a single-centre laboratory study examining a total of 140 SARS-CoV-2 PCR-positive respiratory swab samples, 70 Omicron-BA.1 and 70 Omicron-BA.2, as well as 52 SARS-CoV-2 PCR-negative swabs collected from March 8th until April 10th, 2022. One test did not meet minimal criteria for specificity. In an assessment of the analytical sensitivity in clinical specimen, the 50% limit of detection (LoD50) ranged from 4.2 × 10^4^ to 9.2 × 10^5^ RNA copies subjected to the RAT for Omicron-BA.1 compared to 1.3 × 10^5^ to 1.5 × 10^6^ for Omicron-BA.2. Overall, intra-assay differences for the detection of Omicron-BA.1-containing and Omicron-BA.2-containing samples were non-significant, while a marked overall heterogeneity among the five RATs was observed. To score positive in these point-of-care tests, up to 22-fold (LoD50) or 68-fold (LoD95) higher viral loads were required for the worst performing compared to the best performing RAT. The rates of true-positive test results for these Omicron subvariant-containing samples in the highest viral load category (Ct values < 25) ranged between 44.7 and 91.1%, while they dropped to 8.7 to 22.7% for samples with intermediate Ct values (25–30). In light of recent reports on the emergence of two novel Omicron-BA.2 subvariants, Omicron-BA.2.75 and BJ.1, awareness must be increased for the overall reduced detection rate and marked differences in RAT performance for these Omicron subvariants.

**Supplementary Information:**

The online version contains supplementary material available at 10.1007/s00430-022-00752-7.

## Introduction

During the ongoing COVID-19 pandemic new variants and subvariants of SARS-CoV-2 continue to emerge. Test strategies based on the detection of either viral nucleic acids, primarily by quantitative reverse transcription polymerase chain reaction (qRT-PCR), or of the viral nucleocapsid protein by rapid antigen tests (RATs) contribute to COVID-19 diagnosis and the control of SARS-CoV-2 transmission. This is exemplified by the recent management of the 5th pandemic wave caused by Omicron-BA.1 in Hong Kong [1]. A prerequisite for effective health care interventions is a good sensitivity (> 80%) and high specificity (> 97%) of such RATs fulfilling the minimal criteria set by the Word Health Organization (WHO), especially for RATs recommended for layman’s use [2]. The sensitivity of RATs has been demonstrated to underlie a huge inter-test variability, ranging from 0 to 98.6% [3–6]**.** Importantly, RATs can also show pronounced differences in intra-assay comparisons of sensitivity for the detection of different variants of concern (VoCs) of SARS-CoV-2 [7–11]. Thus, it is important to re-evaluate commercially available RATs on a regular basis by independent laboratories to identify those that still fulfill the WHO performance criteria once a new VoC is starting to dominate the pandemic.

We and others have examined the sensitivity of a number of RATs to detect different VoCs documenting a highly variable inter- and intra-test performance [4, 6–10, 12–20]. With the appearance of the Omicron sublineages BA.1 and BA.2 in late 2021 and early 2022 [21, 22], more mutations in the spike protein, but also in the nucleocapsid protein have been reported [23, 24]. In Germany, the currently available RATs for layman’s use have, to a limited extent, been re-analyzed by the Paul-Ehrlich Institute (PEI) in early 2022 [25]. They concluded that the majority of RATs recognize the Omicron-BA.1 VoC based on their evaluation of a total of four respiratory sample pools and six tissue culture samples as well as theoretical considerations of antibody’s presumed binding sites in a so called “bridging approach” [26]. Recently, the European Commission has updated their information on COVID-19 antigen tests [27]. However, continued evaluations by independent laboratories using sufficient numbers of respiratory swabs from patients are pertinent, in particular with the rapidly evolving subvariants of Omicron. Thus far, only a few studies have addressed this [28–31]. While Omicron-BA.5 is currently dominating the pandemic, recent reports on the emergence of two novel BA.2 subvariants, Omicron-BA.2.75 and BJ.1 [32–35], have alerted the biomedical community to a potential recurrence of this earlier Omicron variant. The aim of our current study was to perform a side-by-side comparison of the diagnostic performance of five commercially available RATs using respiratory samples from patients infected with either Omicron-BA.1 or -BA.2.

## Materials and methods

### Respiratory swabs

Swab specimens were collected by trained medical personnel from patients at COVID-19 testing centers, nursing homes, regional hospitals, and family practices. Flocked Sigma-Transwabs^®^ with 1 or 2 ml Amies Transport Medium (Medical Wire & Equipment Co Ltd; Corsham, UK) were used for this study. No information about vaccination status of individuals, previous infections, presenting symptoms, clinical course or the sampling site in the upper respiratory tract were available. Samples were initially submitted to Labor Becker MVZ GbR in Munich, Germany, a regional diagnostic laboratory, where samples were tested for SARS-CoV-2 RNA by qRT-PCR and subsequently characterized by variant-specific PCR as either Omicron-BA.1 or -BA.2. Samples being detected as positive in this “screening PCR” were randomly included in the study, depending on the availability of a sufficient sample volume and covering naturally occurring viral loads, and analyzed the latest at 24 h after sample collection. Patient specimens in liquid transport medium with the potential for protein denaturation were excluded from the study. Original respiratory swabs and transport media were stored at 2–8 °C for up to 48 h, until samples were inactivated and SARS-CoV-2 RAT evaluation was performed. Due to complex logistics for swab transport, we deviated at times from the recommended procedure because we previously observed that short-term storage at 2–8 °C has no significant impact on the outcome of the test result [15]. Formally, we cannot exclude though that for some of the RATs this may have impacted their performance. A total of 140 PCR-positive (Omicron-BA.1: 70 samples, Omicron-BA.2: 70 samples) respiratory samples were analyzed. The study was conducted in the period 8th of March until 10th of April 2022.

### SARS-CoV-2 rapid antigen tests

The method used in this study is an internationally accepted procedure, in which pre-defined aliquots of each sample need to be completely absorbed using the specimen collection device, e.g. swab, provided with the respective RAT. In this study, 50 µl of the available virus-containing virus transport medium (VTM) solution was completely absorbed. The binary results in this RAT study (“positive” or “negative”) are plotted relative to “RNA copies subjected to test”. For a detailed protocol, please refer to [10]. Trained personnel eluted the soaked swabs in supplied assay buffer following the manufacturer's instructions for processing. Only the use of VTM and storage time prior to testing partially deviated from the manufacturer's instructions (see Suppl. Table 1). RAT reading was performed by experienced and trained personnel blinded to the PCR result under constant light conditions after 15 min incubation. Two visible test lines were recorded as “positive”. In the absence of a visible control line, tests were repeated when possible and the result otherwise was scored as “invalid”.

In detail, the following five RATs were included in the study (for detailed test characteristics, see Table 1 and Suppl. Table 1): Lungene-COVID-19 Antigen Rapid Test Cassette (Hangzhou Clongene Biotech Co.) (“Clongene”), Nadal COVID-19 Ag Test (test cassette) (nal von minden GmbH) (“nal von minden”), Novel Corona Virus (2019-nCoV) Antigen Test Kit (Colloidal Gold Immunochromatography) (Glallergen Co.) (“Glallergen”), InstantSure COVID-19 Ag CARD (Suzhou Soochow University Saier Immuno Biotech Co., Ltd.) (“Saier”) and EGENS Sars-CoV-2 Antigen Rapid Test (Nantong Egens Biotechnology Co.) (“Egens”).

**Table 1:** General information on the five RATs used in this study

Study name	Clongene	nal von minden	Glallergen	Saier	Egens
Manufacturer	Hangzhou Clongene Biotech Co	nal von minden GmbH	Glallergen Co	Suzhou Soochow University Saier Immuno Biotech Co., Ltd	Nantong Egens Biotechnology Co
Authorized representative	Shanghai International Holding Corp.GmbH	–	Osmunda Medical Technology Services GmbH	ZWL GmbH	Shanghai International Holding Corp. GmbH
Test name	Lungene-COVID-19 Antigen Rapid Test Cassette	Nadal COVID-19 Ag Test (test cassette)	Novel Corona Virus (2019-nCoV) Antigen Test Kit (Colloidal Gold Immunochromatography)	InstantSure COVID-19 Ag CARD	EGENS Sars-CoV-2 Antigen Rapid Test
Device identification (European Commission)	1610	2848	2695	3015	1573
BfArM Test-ID	AT079/20	AT021/20	AT755/21	AT830/21	AT425/21
Ref. No	ICOV5002-B025	243103 N-20	600,010	n.a	n.a
HSC common list (RAT)^a^ [27]	Yes	No	Yes	Yes	Yes
PEI evaluated (last update 02.07.2022)^b^ [25, 26]	Yes, but LOT 2,020,110,090	Yes, but AT470/20, REF 243104D-20	Yes, but Ref. No 600008	Yes	Yes
Bridging-evaluation^c^ [26]	Yes	Yes	Pending	Yes	Yes
Antibody used	Monoclonal	Monoclonal	Monoclonal	Monoclonal	SARS-CoV-2 antibodies
Detected antigen	Nucleocapsid protein	Nucleocapsid protein	Nucleocapsid protein	Nucleocapsid protein	SARS-CoV-2 antigen

*n.a.* not available

^a^[24–27]

^b^[26]

Of the five RATs studied, four are listed in the HSC common list of the European Commission [27, 36] (except nal von minden), but all had been previously evaluated by the PEI (nal von minden and Glallergen with a different product version/REF number; see [24, 26]). The “bridging evaluation” performed by the PEI rated four of the RATs as suitable for the detection of the Omicron VoC, only for the Glallergen test this information was still pending at the time of writing the manuscript [25].

### PCR screening, variant-specific PCR and quantitative viral load determination

It was ensured that a time interval of 24 h was not exceeded between swab collection and determination of the viral load. In the retrospective laboratory study, PCR-positive samples from the Becker MVZ GbR laboratory were analyzed, which were identified using the “Munich Extraction Protocol” [37]. After determination of the Omicron sublineage by variant-PCR (modified version of the COVID-19 direct RT-PCR kit (FRIZ Biochem GmbH, Neuried, Germany)), samples containing Omicron-BA.1 or -BA.2 were sent to the Max von Pettenkofer Institute, where quantification by Roche Cobas SARS-CoV-2 E-gene reaction on a Cobas 6800 system (Roche, Mannheim, Germany) was performed under routine diagnostic laboratory conditions. For further details of this methodology, please refer to [10, 15, 38]. Until RAT evaluation swab samples were stored at 2–8 °C for at maximum an additional 24 h.

### RAT specificity

The following approach was chosen for testing the specificity of RATs: In brief, healthy volunteers were swabbed using naso-/oropharyngeal swabs (eSwab™ (Copan Diagnostics, Murrieta, California, USA)). Each 100 µl of transport medium was combined into pools containing a maximum of nine subjects per pool and pseudonymously tested for the presence of SARS-CoV-2 RNA using Xpert Xpress SARS-CoV-2 run on the GeneXpert System (Cepheid Inc., Sunnyvale, California, USA). If the PCR result was negative, all individual respiratory samples from this pool were anonymized. Subsequently, within 3 h after collection of the PCR swab, the five RATs were analyzed using fresh swab specimens, collected in parallel from the PCR-negative pool participants, according to the manufacturer's instructions under the supervision of trained laboratory personnel.

### Statistical analyses

Statistical analysis was performed in R version 4.1.2. Binomial confidence intervals for sensitivities and specificities were computed using the Wilson score interval. To further analyze analytical sensitivities, we used logistic regression, with viral loads and RNA copy numbers subjected to the test as independent and test outcomes as the dependent variable, yielding detection probabilities for each viral load level.

## Results

### Evaluation of RAT specificity

In light of the current Omicron subvariant waves, we sought to evaluate the performance of five different RATs, four of which have been positively evaluated by the PEI to detect Omicron-BA.1 with sensitivity fulfilling regulatory requirements [25–27]. First, the specificity was determined using nasopharyngeal swabs of 52 SARS-CoV-2 PCR-negative healthy volunteers (Table 2). The specificity ranged between 96.15 and 98.08%. Thus, the WHO requirement for a specificity > 97% [2] was fulfilled for all RATs except for the Glallergen test.

**Table 2:** Determination of assay specificity for five qualitative SARS-CoV-2 rapid antigen tests using SARS-CoV-2 PCR-negative respiratory swabs from adults

Assay	Specificity (%)	95% CI	True negative/total
Clongene	98.08	89.88–99.90	51/52
nal von minden	98.08	89.88–99.90	51/52
Glallergen	96.15	87.02–98.94	50/52
Saier	98.08	89.88–99.90	51/52
Egens	98.08	89.88–99.90	51/52

Binomial confidence intervals were computed using the Wilson score interval

### Analytical sensitivity of RATs for detecting Omicron-BA.1 and -BA.2

**Fig. 1:**
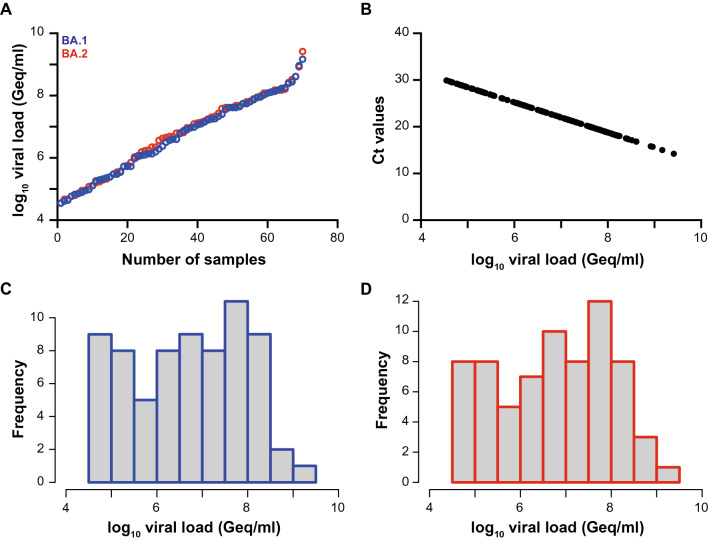
SARS-CoV-2 viral load distribution of respiratory samples included in the study. **A** Shown is the log10 viral load (Geq/ml) of 70 SARS-CoV-2-positive Omicron-BA.1 (blue) and 70 SARS-CoV-2-positive Omicron-BA.2 (red) patient samples, sorted by ascending magnitude of the viral load from left to right. Each dot indicates one patient and the sample’s ID is indicated. **B** Shown is the correlation of the viral loads (Geq/ml) for both Omicron-BA.1 and -BA.2 to the Ct-values, which were obtained with the Cobas 6800 system. **C** Depicted is the histogram of the viral load distribution for Omicron-BA.1 by categorization of samples into defined log10 viral load ranges. Each histogram bar indicates the number of samples in the respective viral load range. **D** Depicted is the histogram of the viral load distribution for Omicron-BA.2 by categorization of samples into defined log10 viral load ranges. Each bar indicates the number of samples in the respective viral load range

Next, we analyzed viral loads in 140 PCR-positive nasal/nasopharyngeal swabs of which 70 were classified as Omicron-BA.1 and 70 as -BA.2, respectively. Viral loads ranged between 35,432 Geq/ml and 1,473,100,589 Geq/ml for Omicron-BA.1 (median: 6,765,509 Geq/ml) and 35,432 Geq/ml and 2,607,495,346 Geq/ml for Omicron-BA.2 (median: 6,422,316 Geq/ml), respectively (Fig. 1). Thus, the median and range of viral loads were comparable for Omicron-BA.1 and -BA.2-containing respiratory samples. Since Ct values vary between different PCR devices, we converted the former results to viral loads presented as Geq per ml to be independent of the respective method (Fig. 1B).

We then evaluated the analytical sensitivity of the five RATs for Omicron-BA.1 and -BA.2 (Table 3 and Table 4). Clongene showed similar sensitivities for Omicron-BA.1 and -BA.2, namely 67 and 56.5%, respectively. No difference between Omicron-BA.1 and -BA.2 could be detected for nal von minden and Saier—the sensitivity for both Omicron-BA.1 and -BA.2 was 58.6% for nal von minden and 55.7% for Saier, respectively. The sensitivity of Glallergen was 48.6% (Omicron-BA.1) and 50% (Omicron-BA.2). Egens detected the lowest percentage of PCR-positive samples for both Omicron subvariants (Table 3 and Table 4).

**Table 3:** Determination of assay sensitivity for five SARS-CoV-2 rapid antigen tests in SARS-CoV-2 PCR-positive respiratory swabs classified as Omicron-BA.1

Omicron-BA.1			
Assay	Sensitivity (%)	95% CI	True positive/total
Clongene	67.14	55.50–77.00	47/70
nal von minden	58.57	46.88–69.37	41/70
Glallergen	48.57	37.25–60.05	34/70
Saier	55.71	44.08–66.75	39/70
Egens	37.68	27.18–49.48	26/69

Binomial confidence intervals were computed using the Wilson score interval

**Table 4:** Determination of assay sensitivity for five SARS-CoV-2 rapid antigen tests in SARS-CoV-2 PCR-positive respiratory swabs classified as Omicron-BA.2

Omicron-BA.2			
Assay	Sensitivity (%)	95% CI	True positive/total
Clongene	56.52	44.79–67.57	39/69
nal von minden	58.57	46.88–69.37	41/70
Glallergen	50.00	38.60–61.40	35/70
Saier	55.71	44.08–66.75	39/70
Egens	35.71	25.50–47.41	25/70

Binomial confidence intervals were computed using the Wilson score interval

**Fig. 2:**
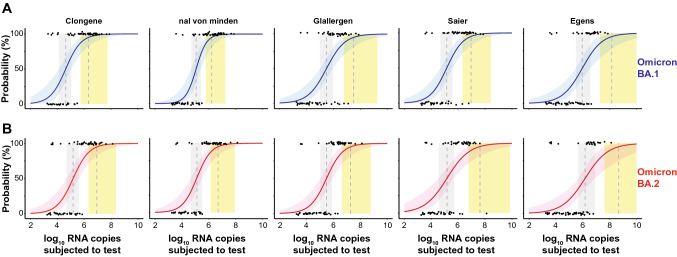
Limit of detection analyses of PCR-positive SARS-CoV-2 patient samples for five SARS-CoV-2 RATs. **A** top panels: Omicron-BA.1 dataset is shown in blue. **B** bottom panels: Omicron-BA.2 dataset shown in red. The log10 RNA copies subjected to the test on the x-axis was plotted against a positive (+ 1) or negative (0) test outcome on the y-axis. For readability of the figure, slight normal jitter was added to the *y* values. Red/blue curves show logistic regressions of the viral load on the test outcome; vertical dashed lines indicate log viral loads at which 50% (LoD50) and 95% (LoD95), respectively, of the samples are expected positive based on the regression results

Next, we determined the 50% (dotted line in grey vertical area) and 95% (dotted line in yellow vertical area) limits of detection (LoD) based on a logistic regression model [10, 12] (Fig. 2A—Omicron-BA.1; Fig. 2B—Omicron-BA.2). The LoD50 and LoD95 values for Clongene equaled 42,009 and 2,082,586 RNA copies for Omicron-BA.1 (Fig. 2A, first panel), respectively. Interestingly, LoD50 and LoD95 values were 3.5 and 4.1-fold higher for Omicron-BA.2 with 148,056 and 8,598,385 RNA copies, respectively (Fig. 2B, first panel).

Nal von minden had LoD50 and LoD95 values for Omicron-BA.1 with 113,386 and 1,607,971 RNA copies, respectively, which were threefold higher or comparable to Clongene. The LoD50 and LoD95 values for Omicron-BA.2 were up to threefold higher compared to Omicron-BA.1, reaching 127,937 and 4,885,350 RNA copies, respectively. In addition, nal von minden was slightly superior in detecting Omicron-BA.2 compared to Clongene (Fig. 2A, B, second panel). Glallergen showed sevenfold and 15-fold higher LoD values compared to Clongene with 296,442 (LoD50) and 31,175,938 RNA copies (LoD95) for Omicron-BA.1 (Fig. 2A, third panel) and only twofold higher LoD values for Omicron-BA.2 with 301,671 (LoD50) and 18,414,042 RNA copies (LoD95) (Fig. 2B, third panel). Here, detection of both Omicron-BA.1 and -BA.2 was comparable for the Glallergen tests. Compared to Clongene, the RAT from Saier had up to fourfold (Omicron-BA.1) and up to fivefold (Omicron-BA.2) higher LoD values, yielding 141,383 (LoD50) and 9,182,132 RNA copies (LoD95) for Omicron-BA.1 (Fig. 2A, fourth panel) and 150,310 (LoD50) and 42,318,341 RNA copies (LoD95) for Omicron-BA.2 (Fig. 2B, fourth panel). In addition, the difference in LoD95 values between Omicron-BA.1 and Omicron-BA.2 was 4.6-fold.

Among the five RATs analyzed, the performance of Egens was worst for both Omicron-BA.1 and -BA.2 (Fig. 2A, B, fifth panel). The LoD50/LoD95 values were increased by 22- and 68-fold for Omicron-BA.1 and 10- and 54-fold for Omicron-BA.2 compared to Clongene, respectively. The viral loads for the LoD50/LoD95 values were 918,552 and 142,176,897 RNA copies for Omicron-BA.1, respectively, and 1,492,762 (LoD50) and 463,200,407 RNA copies (LoD95) for Omicron-BA.2, respectively (Fig. 2A, B, fifth panel). Furthermore, an up to 3.3-fold difference in LoD95 values between Omicron-BA.1 and -BA.2 was observed for Egens. It is of particular note that, although differences in LoD values between Omicron-BA.1 and -BA.2 were noted, the LoD50 and LoD95 values were not significantly different for these two Omicron subvariants among each of the five RATs investigated. In summary, the overall analytical sensitivity of three RATs for the detection of Omicron-BA.1 and -BA.2 was largely comparable (Clongene, nal von minden, Saier), whereas the other two RATs (Glallergen, Egens) showed a considerably reduced sensitivity.

### Comparative, Ct value-stratified evaluation of analytical RAT sensitivity

Similar to our previous data [10], we next thought to compare our results to those reported by Puyskens et al. and Scheiblauer et al*.* [4, 39] (Table 5). This enabled us to score our results based on the Ct/Cp categories < 25, 25–30 and > 30. Except for nal von minden, all other tests are also listed in the EU Common list of COVID-19 antigen tests [27]. In line with our previous analyses, the overall sensitivities were comparable for both Omicron-BA.1 and -BA.2. Interestingly, the overall sensitivity for Clongene and nal von minden was superior to those reported for “Non-Omicron” VoC samples. For the others, the overall sensitivity dropped by 2.5-fold on average.

**Table 5:** Comparative evaluation of the analytical sensitivity of five SARS-CoV-2 rapid antigen tests stratified for Ct/Cp value ranges based on studies by the Paul-Ehrlich-Institute (“non-Delta/non-Omicron”*) and the current study for respiratory samples containing Omicron-BA.1 and -BA.2

	Sample size	Ct < 25 (%)	Ct 25–30 (%)	Ct > 30 (%)	Overall sensitivity (%)
Clongene					
Non-Delta/non-Omicron^a^	n.a	94.4	34.8	0.0	50.0
Omicron-BA.1	70	91.1	21.7	50.0	67.1
Omicron-BA.2	70	76.1	18.2	0.0	56.5
nal von minden					
Non-Delta/non-Omicron^a^	n.a	83.3	13.0	0.0	36.0
Omicron-BA.1	70	86.7	8.7	0.0	58.6
Omicron-BA.2	70	76.6	22.7	0.0	58.6
Glallergen					
Non-Delta/non-Omicron^a^	n.a	100.0	100.0	60.0	92.0
Omicron-BA.1	70	71.1	8.7	0.0	48.6
Omicron-BA.2	70	68.1	13.6	0.0	50.0
Saier					
Non-Delta/non-Omicron^a^	n.a	100.0	100.0	80.0	96.0
Omicron-BA.1	70	80.0	13.0	0.0	55.7
Omicron-BA.2	70	74.5	18.2	0.0	55.7
Egens					
Non-Delta/non-Omicron^a^	n.a	100.0	100.0	50.0	90.0
Omicron-BA.1	70	52.3	13.0	0.0	37.7
Omicron-BA.2	70	44.7	18.2	0.0	35.7

*n.a.* not available

^a^[25, 27]

In the highest viral load category with Ct/Cp < 25, Clongene and nal von minden had rather similar sensitivities as already reported for “Non-Omicron” VoC samples. In contrast, the sensitivities for Glallergen, Saier and Egens were reduced up to 2.2-fold, scoring only 80% down to 44.7% positive samples within this high viral load category (Table 5). The intermediate viral load category, reflected by Ct/Cp values ranging between 25 and 30, showed already more pronounced differences for the Omicron-BA.1/-BA.2 samples: While Clongene and nal von minden had rather comparable sensitivities to the “Non-Omicron” samples, ranging from 8.7 to 22.7%, the other three RATs had about five- to ten-fold lower sensitivities compared to “Non-Omicron” samples, with positive rates ranging between 8.7 and 18.2%. Similar to our previous study [10], samples with Ct/Cp values > 30 were generally not detected with a single exception for Clongene with an Omicron-BA.1-positive sample. In summary, re-evaluation of RATs is highly dependent on the circulating VoCs and should not rely on the previously published analyses with respiratory samples containing previous VoCs.

## Discussion

At the beginning of 2022, the SARS-CoV-2 VoC of the Pangolin lineage B.1.1.529 (Omicron) prevailed in Germany, displacing the previously predominant Delta VoC. Being the most transmissible variant up to this point, the Omicron-BA.1 wave led to the highest incidences in Germany up to now during the COVID-19 pandemic. While the sublineage Omicron-BA.1 was globally dominant, it was rapidly replaced from March 2022 onwards by the apparently even more contagious Omicron-BA.2 sublineage. This study was conducted in early 2022 when Omicron-BA.1 and Omicron-BA.2 were still dominating in Germany. While Omicron-BA.4 evolved, but never became a dominant VoC on a global scale, Omicron-BA.5 has been responsible for the majority of SARS-CoV-2 infections from June 2022 onwards [40]. Currently, the Omicron-BA.5 subline BF.7 is circulating in Europe and increasing cases with Omicron-BA.2.75 and the BA.2-derived BJ.1 are noted worldwide [41]. Details on the performance of RATs for infections caused by Omicron-BA.4 and -BA.5 are in progress but were unfortunately not in the scope of the current investigation. Nevertheless, our current study conducted with Omicron-BA.2 dominating in early 2022 might become more relevant once again due to the rapid evolution of different Omicron subtypes, some specific ones derived from BA.2.

Changes in the virus’ characteristics including also immune escape, in addition to the parallel withdrawal of contact-reducing measures in Germany and the resulting behavioral change in the general population led to a further increase in the number of reported infections [41]. During this period, RATs conducted in official COVID-19 testing centers were an integral part of the country's pandemic management.

The nucleocapsid protein of Omicron-BA.1 shows four mutations compared to the wild-type virus at amino acid positions P13L, DEL31/33, R203K and G204R, Omicron-BA.2 has an additional mutation at S413R. The two Omicron sublineages BA.1 and BA.2 hardly differ in terms of their, on average, less severe clinical manifestation and susceptibility to antibody-mediated neutralization [43–45], yet additional mutations in the spike protein of Omicron-BA.2 seem to be associated with higher infectivity [46, 47]. The currently circulating Omicron-BA.5 has similar mutations in the nucleocapsid as Omicron-BA.2 [48]. Thus, we speculate that our results for Omicron-BA.2 might in part predict those for Omicron-BA.5. In addition to these phenotypic changes, mutations in other functional viral proteins also occur in Omicron and its sublineages, possibly underlying the altered pathogenesis [49, 50]. It has been suggested that a different cell tropism and entry mechanisms may account for different infection kinetics with shortened incubation periods in Omicron compared to earlier VoCs [51–55].

Pre-existing immunity, vaccine- or infection-induced, with the presence of anti-spike antibodies in the swab sample on the one hand, but also different levels of nucleocapsid protein relative to viral RNA loads due to modified replication and pathogenesis on the other hand, may impact on the clinical performance of RATs. In the context of performance evaluation of RATs, it is not yet clear to what extent these factors need to be taken into account in the RAT assessment of VoCs as well as the changing population’s immunity. Potentially, also the nucleocapsid protein, which is the target of nearly all RATs, may accumulate additional mutations in future VoCs that could affect the performance of individual tests. With the emergence of the recent Omicron-BA.4 and -BA.5 subvariants [56], mutations already exist in regions of the nucleocapsid protein that were previously considered as highly conserved. Test manufacturers still protect information regarding the binding sites of their RAT antibodies based on intellectual property claims. Consequently, a constant laboratory-centered re-evaluation of RATs is necessary when newly emerging SARS-CoV-2 variants start circulating since a change of the antigenic epitopes or clinical characteristics that are relevant for testing cannot be excluded.

Although various independent studies comparing Delta with Omicron VoCs show partly contradictory results, there is still appreciation of the potential influence of RAT extraction buffers in connection with the preexisting immunity of infected individuals [57]. Likewise, we are not aware of studies investigating how different concentrations of SARS-COV-2-specific antibodies in pooled study samples influence the sensitivity of RATs.

In the current report, the S413R mutation present in the Omicron-BA.2 nucleocapsid protein does not seem to impair binding of the specific antibodies used in the five RATs evaluated compared to Omicron-BA.1. Similarly, differences in pre-existing antibodies in patients infected with Omicron-BA.1 or -BA.2 that may bind viral particles and thus hamper nucleocapsid recognition by RAT antibodies, seem not to have had a marked influence on test performance.

In comparison to our previous study in which a marked VoC-dependent decrease in the detection of Omicron-BA.1 compared to Delta was observed in 7 out of 9 RATs [10], no significant intra-test specific differences between LoD50/LoD95 values were observed between Omicron-BA.1 and -BA.2. Nevertheless, it is remarkable that the overall sensitivities of the five RATs used in this study also showed considerable inter-test variability and an impaired detection rate for Omicron-containing respiratory samples compared to PEI evaluations [26]. The RATs Glallergen (92% overall sensitivity), Saier (96% overall sensitivity) and Egens (90% overall sensitivity), that were rated “very good” in the PEI evaluation for “non-Delta/non-Omicron” samples, performed worse in the overall sensitivity of our Omicron-based evaluation (Omicron-BA.1/-BA.2: 49–50%, 56%, and 36–38%, respectively) compared to the RATs Clongene and nal von Minden, which were rated worse in the PEI evaluation with a 50 and 36% overall sensitivity, respectively. The latter even achieved higher sensitivities (57–67% and 59%, respectively) in our study with Omicron-BA.1 and -BA.2 than for the “non-Delta/non-Omicron” evaluation by the PEI [25, 26].

This underlines the need for internationally harmonized criteria for independent evaluation studies of RATs, such as the ones launched in the meantime by the European Commission [27]. However, these need to be re-evaluated and adapted to meet the constantly changing requirements, namely the ongoing changes in the immunity of the population and antigenic properties of the SARS-CoV-2 nucleocapsid protein. Only this way the usefulness of RATs as a testing strategy to identify acutely infected individuals can be assessed based on well-founded evidence by policy-makers.

## Supplementary Information

Below is the link to the electronic supplementary material.

Supplementary file1 (DOCX 16 KB)
