# Supplementary material for: Variable detection of Omicron-BA.1 and -BA.2 by SARS-CoV-2 rapid antigen tests
Source: Med Microbiol Immunol. 2022 Nov 12;212(1):13–23. doi: 10.1007/s00430-022-00752-7 (PMC9660148; doi:10.1007/s00430-022-00752-7)
Supplement: Supplementary file 1 — Supplementary file1 (DOCX 16 KB) [file 430_2022_752_MOESM1_ESM.docx]

**Supplementary Table 1** Summary of manufacturer specifications and recommendations for the five RATs used in this study

| **Study name** | **Clongene** | **nal von minden** | **Glallergen** | **Saier** | **Egens** |
| --- | --- | --- | --- | --- | --- |
| **Manufacturer** | Hangzhou Clongene Biotech Co. | nal von minden GmbH | Glallergen Co. | Suzhou Soochow University Saier Immuno Biotech Co., Ltd. | Nantong Egens Biotechnology Co. |
| **Test name** | Lungene-Covid-19 Antigen Rapid Test Cassette | Nadal Covid-19 Ag Test (test cassette) | Novel Corona Virus (2019-nCoV) Antigen Test Kit (Colloidal Gold Immunochromatography) | InstantSure Covid-19 Ag CARD | EGENS Sars-CoV-2 Antigen Rapid Test |
| **Recommended material** | Nasal swab, Nasopharyngeal swab, Oropharyngeal swab; direct swab specimen or swab in viral transport media (VTM) | Nasal swab, Nasopharyngeal swab, Oropharyngeal swab; direct swab specimen or swab in viral transport media (VTM; less than 1ml recommended) | Nasal swab | Nasal swab, Nasopharyngeal swab; direct swab specimen or swab in viral transport media (VTM) | Nasopharyngeal swab, Oropharyngeal swab |
| **Intended by the manufacturer for self-testing** | no- intended for professional use | no- intended for professional use | no- intended for professional use | no- intended for professional use | no- intended for professional use |
| **Storage before testing** | Should be processed as soon as possible, but not later than one hour after specimen collection. Specimen collected may be stored at 2-8°C for no more than 24 hours; Tore at -70°C for a long time, but avoid repeated freeze-thaw cycles. | Should be tested immediately after collection. If not tested immediately, swab specimens can be stored in viral transport media without denaturing agents at 2-8°C for 24 hours after collection. | As soon as possible. If not the specimen should be stored in a dry, sterilized and strictly sealed plastic tube immediately. Can be stored for 8h at 2-8°C or -70°C for a long time. | As soon as possible. (Samples are stable within 30 minutes when stored in the sample extraction solution provided with the kit) | n.a. |
| **Sensitivity** | Nasopharyngeal Ct value <33: 98%; Nasopharyngeal Ct value <37: 92%; Nasal Ct value <33: 97.1%; Nasal Ct value <37: 91.4% | Ct 20-30: 97.6%; Ct 20-37: 80.2%; Ct <30: 94.12% | 94.44% | Nasopharyngeal Ct value <33: 97.06%; Nasopharyngeal Ct value <37: 93.68%; Nasal Ct value <33: 96.32%; Nasal Ct value <37: 92.98% | 95.80% |
| **Specificity** | Nasopharyngeal Ct value <33: 99.7%; Nasopharyngeal Ct value <37: 99.7%; Nasal Ct value <33: 99.4%; Nasal Ct value <37: 99.4% | >99.9% | 99.02% | Nasopharyngeal Ct value <33: 99.68%; Nasopharyngeal Ct value <37: 99.68%; Nasal Ct value <33: 99.68%; Nasal Ct value <37: 99.37% | 99.80% |
| **Limit of detection (TCID50)** | 5,7 x 10² TCID50/ml | 2 x 10²^,4^ TCID50/ml | 9.65 TCID50/ml | 160 TCID50/ml | 14.4 TCID50/ml |
| **Limit of detection (protein concentration)** | - | 0.4 ng/ml | - | - | 10 pg/ml |

n.a.: not available
